# Cystic plate approach in laparoscopic cholecystectomy: a consecutive retrospective analysis

**DOI:** 10.3389/fsurg.2024.1487568

**Published:** 2024-12-03

**Authors:** Yanjie Zhou, Le Xiao, Zhulin Luo, Hao Luo, Zhen Tan, Tao Wang

**Affiliations:** ^1^ Department of Hepatobiliary Surgery, Chengdu Medical College, Chengdu, Sichuan, China; ^2^ Department of General Surgery, The General Hospital of Western Theatre Command, Chengdu, Sichuan, China

**Keywords:** cholecystectomy, cystic plate, gallbladder, iatrogenic biliary injury, laparoscopy

## Abstract

**Purpose:**

This study aimed to investigate the safety, feasibility, and possible advantages of the cystic plate approach during laparoscopic cholecystectomy in a retrospective cohort of surgical patients.

**Methods:**

We summarized the key points of the technical approach, retrospectively analyzed the clinical outcomes of 156 patients in the cystic plate approach group from July 2018 to July 2023, and compared the findings with those of 173 cases in the routine approach group from the same period.

**Results:**

We observed no differences in the average stone size, operation time, postoperative hospital stay, conversion rate, complications, or Visual Analog Scale pain scores on the second day of surgery between the two groups (*p* = 0.076, 0.067, 0.278, 1.000, 0.633, and 0.131, respectively). However, intraoperative blood loss, number of clips used, volume of postoperative drainage fluid, and Visual Analog Scale pain scores on the day of surgery in the cystic plate approach group were significantly lower than those in the routine approach group (*p* = 0.000, 0.031, 0.027, and 0.021, respectively).

**Conclusions:**

The cystic plate approach is a safe, feasible, and effective approach that has the advantages of minimal invasiveness with less bleeding and seepage, reduced use of biological clips, and less pain, potentially minimizing the risk of iatrogenic biliary injury.

**Trial registration:**

This study was registered at the International Clinical Trial Registry (ChiCTR2100052860). Registration date: November 6, 2021.

## 1 Introduction

Laparoscopic cholecystectomy (LC) is a common and risky hepatobiliary surgery that may be accompanied by iatrogenic biliary injury (IBI) during surgery (1). Although the overall incidence of IBI during LC has been approximately 0.2%–0.5% in recent years, nearly 2 million people in China undergo LC each year. The catastrophic consequences of IBI make it a significant public health and medical concern that surgeons need to address in every surgery meticulously.

In 1995, Strasberg presented the principle of the critical view of safety (CVS), significantly reducing the incidence of IBI (2). This principle is considered the optimal criterion for preventing IBI in LC worldwide. Following this principle, multiple technical methods have been introduced to enhance the safety of LC. For challenging cases of gallbladders with acute inflammation, techniques such as Rouvière's sulcus-first approach, inside approach to the gallbladder, and cystic artery approach have been reported (3–6). Most patients who undergo cholecystectomy do not have acute cholecystitis. In practice, IBI is more likely to occur in common cases because of repeated attempts by inexperienced surgeons to achieve CVS (2). The most common type of IBI occurs when the common bile duct (CBD) is severed instead of the cystic duct. This misidentification may have resulted from an inappropriate surgical approach (3).

With the widespread application of high-resolution laparoscopy and an updated understanding of biliary tract anatomy, we are looking to improve the treatment results of routine LC by updating traditional techniques, especially for young surgeons. In this study, we present a novel surgical approach, the cystic plate approach (CPA), and evaluate its safety and potential advantages over traditional surgical techniques.

## 2 Methods

### 2.1 Patients

A total of 503 consecutive patients (201 men and 302 women) with gallstones but without acute cholecystitis who consented to undergo LC surgery were enrolled between July 2018 and July 2023 in department of general surgery, The General Hospital of Western Theatre Command. The patients were between 16 and 85 years of age, with right upper abdominal pain or subxiphoid pain and gallbladder stones confirmed by ultrasonography at our center. Patients with CBD dilation were recommended to undergo magnetic resonance cholangiopancreatography to exclude CBD stones (diameter >0.6 cm). Patients over 60 years of age are recommended to undergo pulmonary function tests and arterial blood gas analysis to assess the condition of the lungs. Patients who had a gallbladder wall thickness of more than 0.5 cm, had acute cholecystitis lasting more than 72 h, had a history of percutaneous transhepatic gallbladder drainage, were diagnosed with choledocholithiasis, or were postoperatively diagnosed with gallbladder cancer were excluded from the study.

CPA is a technical update based on a routine approach, and its potential advantages have been discovered through preliminary research. Therefore, after approval by the Ethics Committee of the General Hospital of Western Theatre Command (Approval No: 2021ky118-1), we decided to switch to CPA on December 31, 2020. The routine approach (RA) group included patients enrolled before this date. Written informed consent was obtained from all participants. They underwent the RA procedure, whereas those admitted after this date were enrolled in the CPA group and underwent LC via CPA. The same surgical team of three surgeons performed all operations. The clinical characteristics and perioperative results of the two groups were retrospectively analyzed. This study was registered on the International Clinical Trial Registry website (ChiCTR2100052860). Written informed consent was obtained from all patients. Data were obtained from a retrospective analysis of the patient's charts.

### 2.2 Surgical procedure

#### 2.2.1 CPA

**Figure 1:**
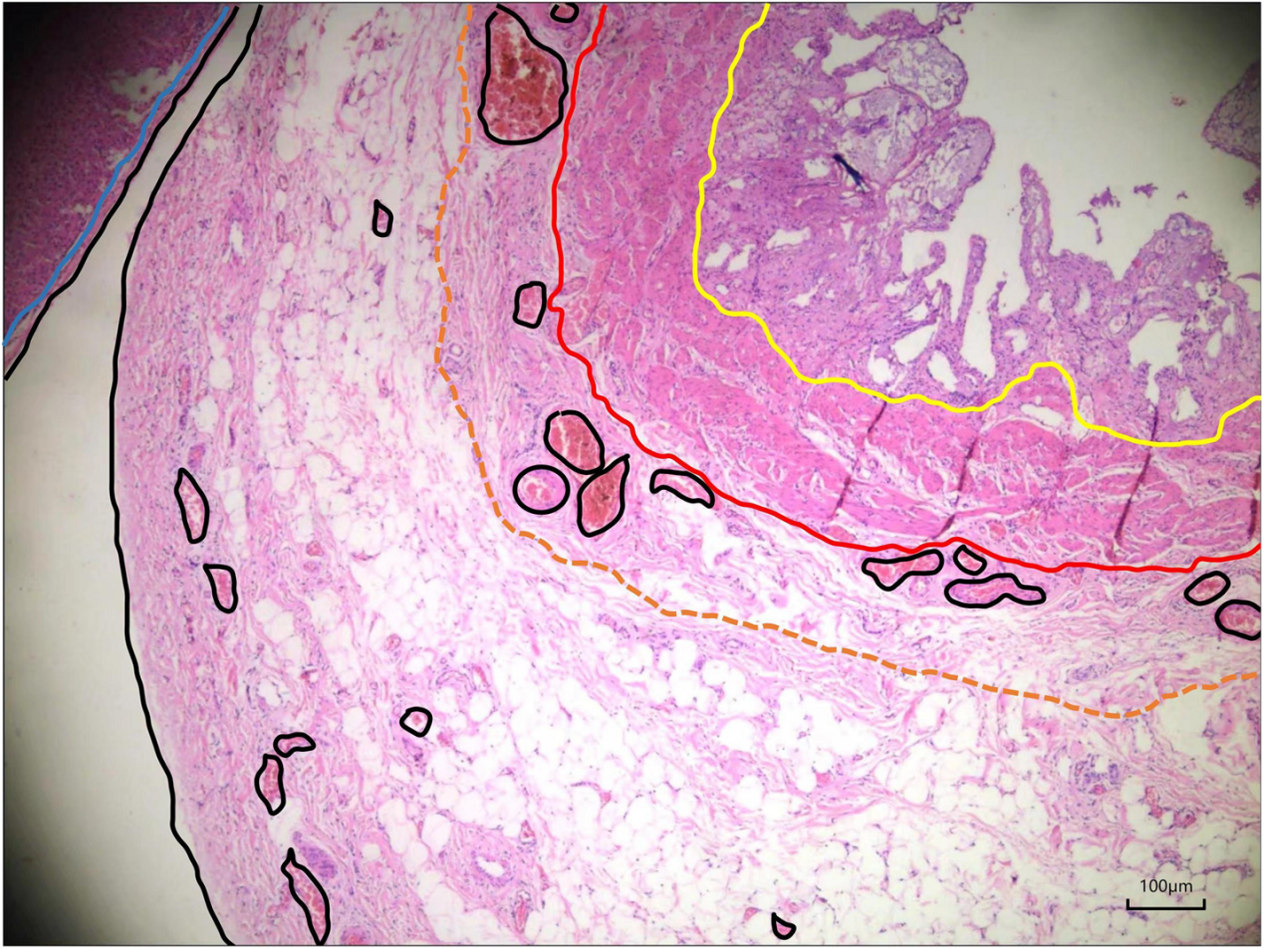
Microscopic view of the gallbladder wall. The free side of the gallbladder wall was divided into the mucosal layer (yellow line), muscularis propria layer (red line), subserosa layer, and serosa layer (black line). The subserosal layer can be divided into the inner layer, which is dominated by fibrous tissue and large vessels, and the outer layer, which is dominated by adipose tissue and small vessels. The brown dotted line shows the dividing line between the inner and outer layers. The cystic plate is the inner subserous layer of the gallbladder wall extending from the hilar plate and adheres to the Laennec membrane (blue line).

**Figure 2:**
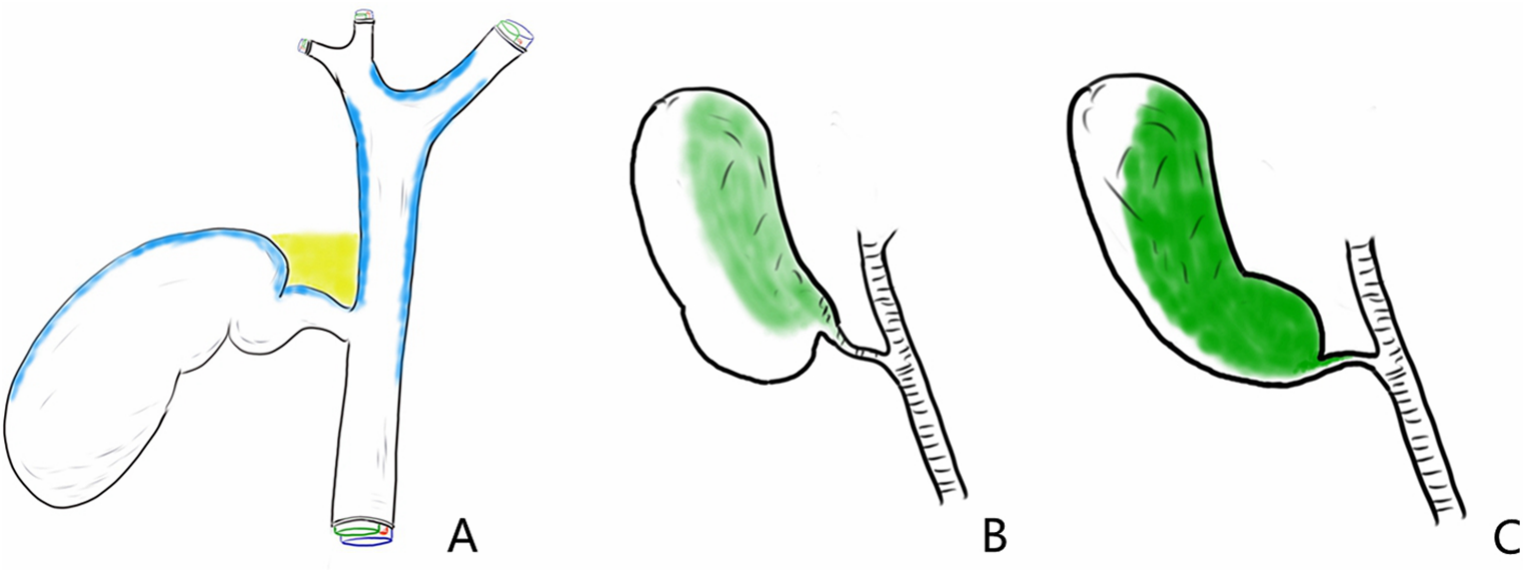
A potential space exists between the cystic and hilar plates. **(A)** Potential gap (marked in yellow) between the cystic and hilar plates (marked in blue). **(B)** Original position of the gallbladder (shown in green as the area of the gallbladder plate attached to the liver). **(C)** The cystic plate is separated from the liver, and the space between the cystic plate and hilar plate is opened away from the common bile duct.

The subserosal layer of the gallbladder wall can be divided into the inner layer, which is dominated by fibrous tissue and large vessels, and the outer layer, which is dominated by adipose tissue and small vessels. The cystic plate is the inner subserous layer that extends from the hilar plate (Figure 1). Theoretically, a potential space exists between the cystic and hilar plates (marked in yellow in Figure 2). The process begins at the hepatocystic triangle, followed by locating and identifying the gallbladder plate and dissecting closely along the gallbladder until it is completely free in the outer subserous layer of the gallbladder wall.

Surgery was performed in the supine position, with the patient's head elevated and inclined toward the left. An observation trocar was inserted into the umbilicus (Supplementary Video 1). Hartmann's pouch was stretched to the lower right to uncover the hepatocystic triangle, and the serosal surface of the gallbladder was opened using electrocautery. The cystic plate was easily identified and dissected near the gallbladder in this area. The separation plane extended along the cystic plate to the gallbladder fundus and cystic duct. During this procedure, the cystic artery is usually identified and clipped. Surgeons pulled the gallbladder and rotated the dorsal side of Hartmann's pouch to the front to further expand the space between the cystic and hilar plates (Supplementary Video 2). The cystic plate was completely separated from the hilar plate, the gallbladder was completely separated from the gallbladder bed, and CVS was confirmed. The cystic duct was clamped 0.5 cm from the CBD with absorbable clips (Lapro-Clip 12 mm, COVIDIEN, USA) (Supplementary Video 3). The gallbladder bed was treated with electrocoagulation. Abdominal drainage was positioned into Morrison's pouch. None of the patients received additional postoperative opioid analgesics. When the total amount of peritoneal drainage fluid was less than 10 ml for 12 consecutive hours, the drainage was removed, and the total amount of postoperative drainage was recorded.

#### 2.2.2 Routine approach

Anesthesia, body position, trocar setting, and postoperative management were the same for RA and CPA. Retrograde cholecystectomy or conventional cholecystectomy was performed, including blunt dissection, by exposing the hepatocystic triangle (7). Blunt separation is often used in conventional procedures to expose the main cystic duct and cystic artery by physically breaking apart the fatty tissue and small ducts. After cutting off the cystic duct and cystic artery in the first step, the gallbladder was separated from the liver bed. Retrograde cholecystectomy, known as the fundus-first or top-down method, involves dissociation of the gallbladder fundus before dissection of Calot's triangle, resulting in good exposure of Calot's triangle and easy confirmation of CVS. This procedure is widely used in cases of chronic or acute inflammation as well as complicated anatomy where a safe plane of dissection is not available.

### 2.3 Statistical analysis

Statistical analysis was performed using SPSS Version 15.0 for Windows (SPSS, Chicago, USA). Continuous data are presented as mean ± standard deviation and were evaluated using Fisher's exact *t*-test or Mann–Whitney test. Categorical data were described using frequency counts and assessed using the chi-square (*χ*^2^) test. Statistical significance was set at *p* < 0.05.

## 3 Results

**Figure 3:**
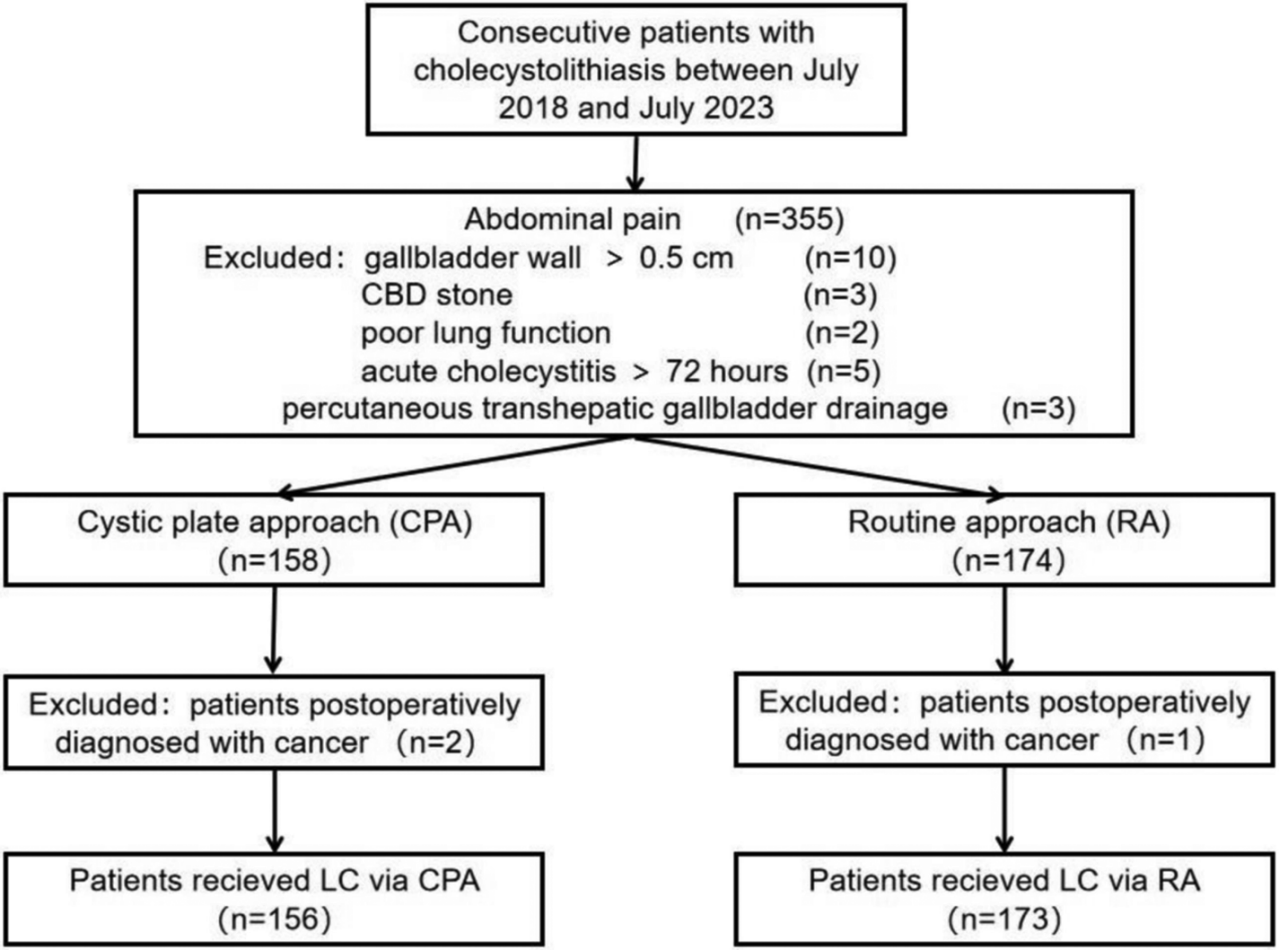
CONSORT diagram of participants assigned to the cystic plate approach (CPA) group vs. the routine approach (RA) group.

A total of 329 cases were retrospectively enrolled in this study, with 156 undergoing CPA and 173 undergoing RA. Five patients were converted to open surgery for hepatic vein injury, stone incarceration in the cystic duct, and cystic duct atresia. No biliary tract injury was observed in any case. The CONSORT diagram for patient enrolment is illustrated in Figure 3. Age, sex, number of stones, average stone size, and history of laparotomy were similar between the CPA and RA groups (Table 1). We observed no differences in the operation time (*p* = 0.067), postoperative hospital stay (*p* = 0.278), conversion to laparotomy (*p* = 1.000), complications (*p* = 0.633), and Visual Analog Scale (VAS) pain scores on the second day of surgery (*p* = 0.131), indicating that CPA was safe and feasible. However, intraoperative blood loss (*p* = 0.000), number of clips used (*p* = 0.031), volume of postoperative drainage fluid (*p* = 0.027), and VAS pain scores on the evening of the day of surgery (*p* = 0.021) in the CPA group were significantly lower than those in the RA group, indicating that CPA is a minimally invasive approach.

**Table 1:** Baseline characteristics of all patients in each group.

Characteristics	CPA (*n* = 156)	RA (*n* = 173)	Magnitude	*p*
Sex			*χ*^2^ = 0.070	0.792
Male	60	69		
Female	96	104		
Age (years)	46.1 ± 13.74	46.86 ± 13.16	t^c^ = −0.502	0.616
BMI	24.01 ± 2.03	24.11 ± 1.91	t = 0.487	0.626
Number of stones			χ^2^ = 0.811	0.368
Single	11	17		
Multiple	145	156		
Stone diameter (mm)	1.5 ± 0.71	1.4 ± 0.62	t = 1.78	0.076
Laparotomy			U^d^ = 0.000	1.000
Yes	2	3		
No	154	170		
Operation time (min)	47.5 (IQR 34.2–55)	45(IQR 28–50)	U = 11,919.5	0.067
Number of clips (*n*)	2 (IQR 2–4)	4 (IQR 2–5)	U = 16,332	0.031
Bleeding volume during the operation (ml)	10 (IQR 10–20)	20(IQR 10–20)	U = 17,882.5	0.000
VAS on day 1^a^	1 (IQR 1–2)	2 (IQR 1–3)	U = 15,431	0.021
VAS on day 2^b^	0 (IQR 0–1)	0 (IQR 0–1)	U = 14,584.5	0.131
Post-operational fluid volume (ml)	15 (IQR 10–20)	15 (IQR 10–40)	U = 11,624	0.027
Complications [*n* (%)]	8 (5.12)	11 (6.36)	χ^2^ = 0.228	0.633
Hospital stay (days)	3 (3,4)	3 (3,4)	U = 14,390	0.278

CPA, cystic plate approach; RA, routine approach; VAS, visual analog scale; IQR, interquartile range.

^a^ Scores were recorded at 21:00 on the surgery day.

^b^ Scores were recorded at 9:00 on the second day of surgery.

^c^ t value in Student *t*-test.

^d^ U value in Mann–Whitney U rank sum test.

## 4 Discussion

**Figure 4:**
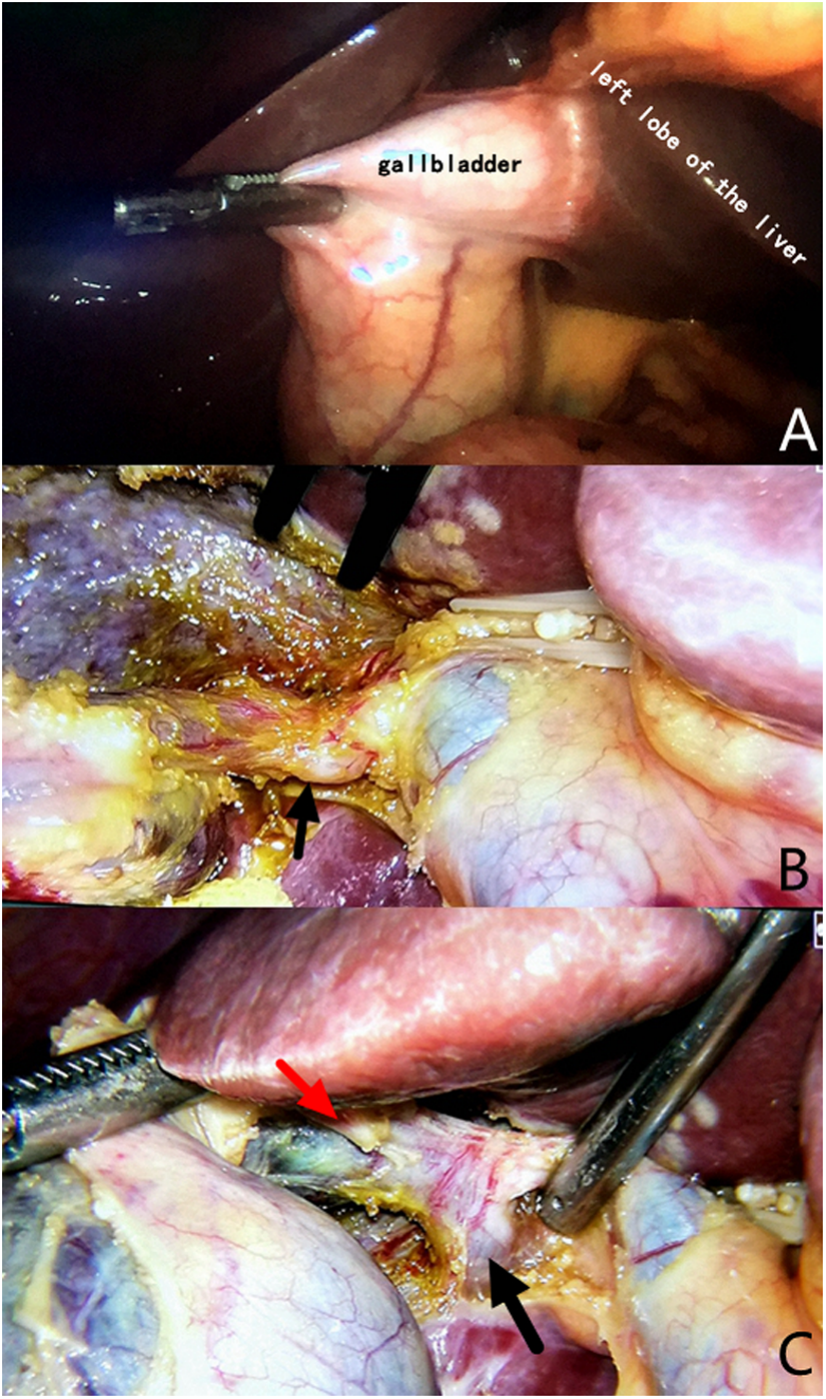
Variations in gallbladder location or cystic duct anatomy. **(A)** The gallbladder is located in the left lateral lobe of the liver. **(B)** Mutated cystic duct (black arrow) with no apparent abnormalities, as observed from the front. **(C)** The mutated cystic duct (red arrow) converges into the accessory right hepatic duct (black arrow), increasing the risk of injury.

**Figure 5:**
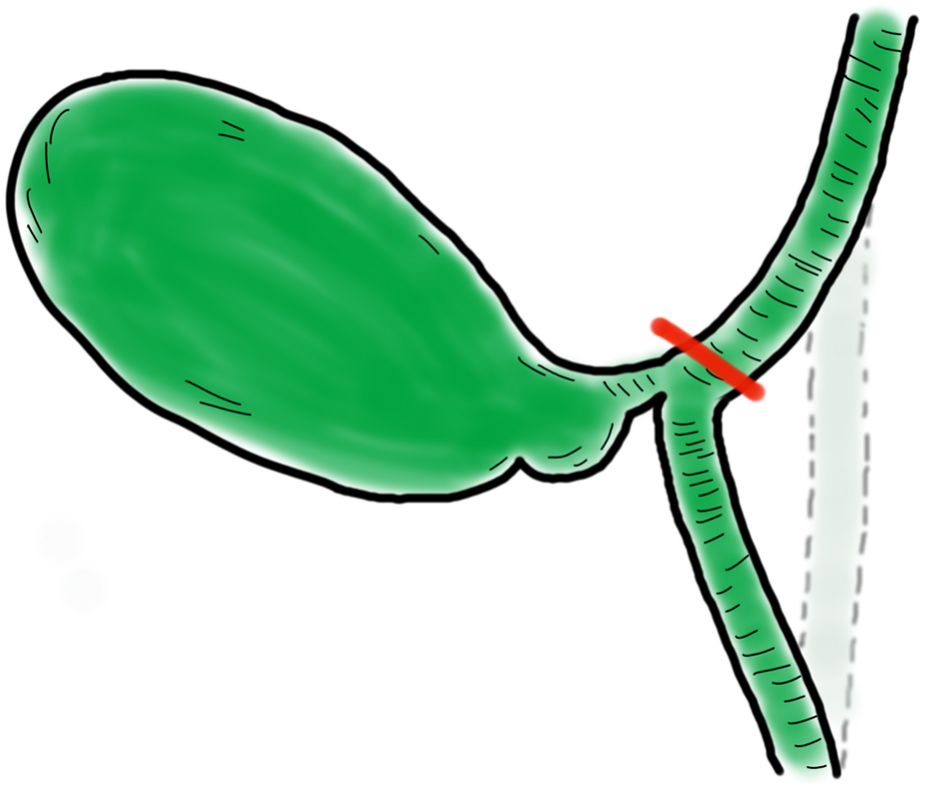
The most common iatrogenic biliary injury. Iatrogenic biliary injury is marked in red, and its original position is represented by the dotted line area.

Since the first LC surgery performed by Mouret in 1987 (8), open cholecystectomy has generally been replaced by LC owing to the advantages of this procedure, such as minimal invasiveness, fast recovery, and less pain (9). Hence, LC has become the first-line treatment for patients with cholecystolithiasis. There is a high incidence of gallbladder stones in China, and IBI continues to be one of the most severe complications that deserves great attention (10). CVS is widely accepted worldwide and has three requirements. First, Calot's triangle must be cleared of fat and fibrous tissue. Second, the lower part of the gallbladder must be separated from the fibrous surface of the liver. Third, only two structures entering the gallbladder should be observed (11). However, achieving CVS safely is challenging, especially in cases with variations in gallbladder location or cystic duct anatomy (Figure 4). For some inexperienced surgeons, IBI occurs when they overpursue CVS through an incorrect approach. The most common IBI occurs when the upper segment of the CBD is pulled too far from its original position due to a short cystic duct or inadequate denudation of the cystic duct (Figure 5). Previously reported techniques have mainly focused on difficult cholecystectomies, such as when purulent or severe acute cholecystitis is present.

With a deep understanding of the anatomical structure of the cystic plate and the application of a high-resolution laparoscopic system, this study aimed to upgrade the traditional technique for treating common non-acute inflammation of the gallbladder to perform LC more safely, easily, and with minimal invasiveness. Chen et al. reported that CPA has the advantage of easy and safe freeing of the target liver pedicle in precise anatomical hepatectomy and biliary surgery, and the cystic plate provides a landmark for the inferior edge in total removal of the gallbladder (12). In our study, we found that the CPA group obtained similar results to the RA group in terms of operation time, postoperative hospital stay, conversion rate, complications, and VAS score on the second day of surgery, suggesting that it is safe and feasible. In traditional techniques, blunt separation or a combination of blunt separation and electrosurgical separation is preferred to avoid bile duct damage. However, blunt separation results in injury to tiny vessels, such as those within the bile duct, general blood vasculature, or lymph duct, which may lead to postoperative bleeding or fluid collection and is a major disadvantage. With the help of a high-resolution camera system, small pipe structures and the cystic plate can be identified and cut using electrical coagulation via the CPA, which may reduce the number of clips required. Precise dissection reduces intraoperative bleeding and damage to microscopic and lymphatic vessels caused by blunt separation. Therefore, this precise dissection reduces intraoperative blood loss and postoperative drainage volumes and relieves postoperative pain without prolonging the operation time.

**Figure 6:**
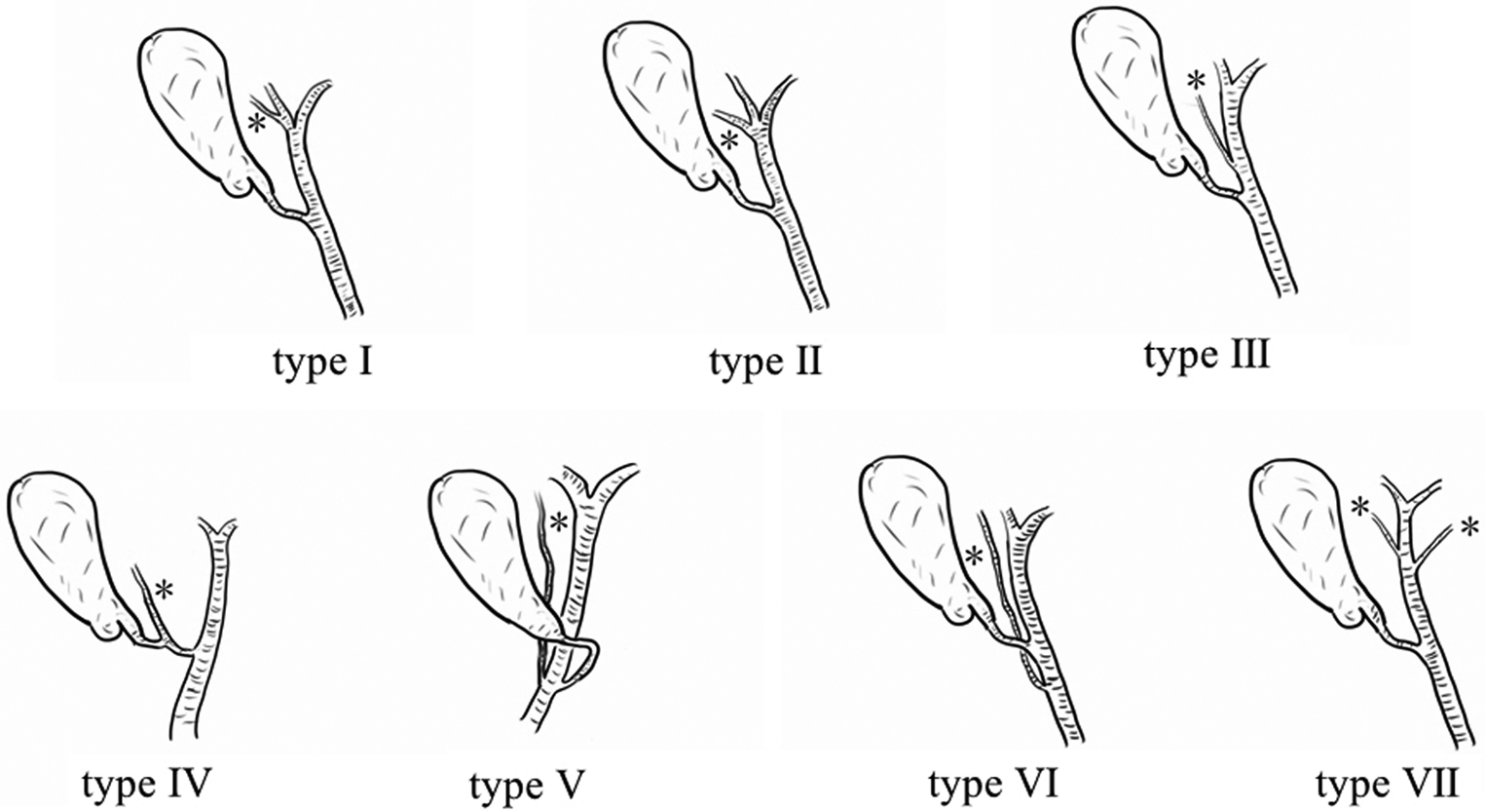
Seven types of accessory bile duct variations. A Type I accessory bile duct converges into the right/left hepatic duct; a Type II accessory bile duct converges into the bifurcation of the left and right hepatic ducts; a Type III accessory bile duct converges into the hepatic duct; a Type IV cystic duct converges into the accessory bile duct; a Type V cystic duct converges into the left side of the common bile duct, and the accessory bile duct converges into the right side of the common bile duct; a Type VI accessory bile duct converges into the common bile duct; and a Type VII double accessory bile duct converges into the hepatic duct. (*Indicates the accessory bile duct.).

The theoretical foundation of CPA is a subserous fibrotic tissue called the gallbladder plate. Dissection should be performed close to the cystic plate on the gallbladder side to protect any other aberrant structures from passing across this potential space. When the concealed space is revealed, the implementation of the CVS becomes more secure and faster. Remarkably, the anatomy and configuration of the cystic duct and CBD showed multiple variations. A better understanding of accessory bile duct variations is conducive to the application of CPA. There are seven types of variations (Figure 6) (13). Type I, II, and VII variations are not easily injured because of their distance from the cystic duct. Variations in types III and V may be damaged because of their proximity to the cystic duct. In contrast, type IV and VI variations may be easily injured because the cystic duct crosses over the accessory hepatic duct. However, even variations in the accessory bile duct with a high risk of injury should originate within the hilar plate, which can be separated from the cystic plate. In addition, the greater and wider use of high-resolution laparoscopy can enhance the identification of fine structures. In theory, CPA may prevent bile duct injury as much as possible and shorten the learning curve of young surgeons. Since the incidence of IBI during LC is about 2–5 per 1,000, the incidence of complications, especially biliary injury, did not show significant differences in this study, and insufficient sample size is the limitation of this study.

Compared with RA, CPA in LC is safe, feasible, and minimally invasive. CPA in LC may be optimal for achieving CVS during surgery for most LCs. However, this preliminary conclusion must be further verified in a multicenter study with a prospective trial approach and within studies with sufficient magnitude to establish this association.

## Data Availability

The original contributions presented in the study are included in the article/Supplementary Material, further inquiries can be directed to the corresponding authors.
